# Grey wolf optimization for sensory-driven fortification of biscuits with moringa leaf and jackfruit seed powders: balancing processing and consumer acceptance

**DOI:** 10.3389/fnut.2026.1779387

**Published:** 2026-04-24

**Authors:** Dhananjay Kumar, Chaman Kumar, Ankit Kumar, Chinmaya Sahoo, Ankit Saini, Kübra Sağlam, Mehmet Ali Şimşek, Seydi Yıkmış, Emad Karrar, Moneera O. Aljobair, Isam A. Mohamed Ahmed

**Affiliations:** 1 Dr. Rajendra Prasad Central Agricultural University, Samastipur, Bihar, India; 2 Icar-National Research Centre on Litchi, Muzzafarpur, Bihar, India; 3 Department of Agronomy, Eternal University, Baru Sahib, Sirmour, Himachal Pradesh, India; 4 Department of Food Engineering, Faculty of Agriculture, Tekirdağ Namik Kemal University, Tekirdag, Türkiye; 5 Department of Software Engineering, Faculty of Engineering and Natural Sciences, Bandırma Onyedi Eylül University, Bandırma, Balıkesir, Türkiye; 6 Department of Food Technology, Tekirdag Namık Kemal University, Tekirdag, Türkiye; 7 Department of Plant Sciences, North Dakota State University, Fargo, ND, United States; 8 Department of Sports Health, College of Sports Sciences and Physical Activity, Princess Nourah Bint Abdulrahman University, Riyadh, Saudi Arabia; 9 Department of Food Sciences and Nutrition, College of Food and Agricultural Sciences, King Saud University, Riyadh, Saudi Arabia

**Keywords:** biscuits, gray wolf optimization, jackfruit seed powder, moringa leaf powder, process parameter optimization

## Abstract

**Introduction:**

This study aimed to optimize the process parameters for developing nutritionally enriched biscuits incorporating *Moringa oleifera* leaf powder (MOLP) and *Artocarpus heterophyllus* (jackfruit) seed powder using Response Surface Methodology (RSM).

**Methods:**

A Box-Behnken design was employed to evaluate the effects of MOLP percentage (10–30%), baking temperature (170–200°C), and baking time (10–14 min) on six critical responses: spread ratio, colour index, hardness, water activity, baking weight loss, and overall sensory acceptability. In addition to RSM-based numerical optimisation, the Grey Wolf Optimisation (GWO) algorithm was used to further optimise the polynomial models generated by RSM, enabling a robust metaheuristic search for global optima and enhancing the reliability of the optimised process conditions.

**Results:**

Numerical optimization yielded an ideal formulation comprising 10% MOLP, 30% jackfruit seed powder, a baking temperature of 193.68°C, and a baking time of 13.97 minutes. The optimized biscuit exhibited a spread ratio of 6.22, colour index of 161.68, hardness of 2500.58 g, water activity of 0.66, baking weight loss of 7.23%, and overall acceptability score of 7.95, with a desirability of 0.828.

**Discussion:**

Validation trials showed strong agreement between predicted and experimental values for most responses (*p* > 0.05), confirming the model’s reliability. These findings demonstrate the potential of underutilized plant ingredients to improve biscuit nutrition while maintaining sensory quality, supporting their use in functional food development.

## 1 Introduction

The word “biscuit” originates from Latin term *Biscoctum*, which means “twice baked.” In the past, biscuits were referred to as a sick man’s diet but at present the people of all ages eat biscuits the most. Biscuits are highly popular and considered one of the most delicious snacks, making them a leading category in the baked goods industry (1). They offer several advantages, including convenience, variety, widespread consumption, and a long shelf life (50). Today, biscuits are often fortified with complex wheat flour or other nutrient-rich ingredients to enhance their nutritional value (2).

This fortification enhances nutritional value while preserving biscuit quality. The Indian biscuit industry flourished in the 20th century, gaining popularity as an affordable, convenient food, especially in Maharashtra, Uttar Pradesh, Andhra Pradesh, Karnataka, and West Bengal. In India, the average biscuit consumption is 2.1 kg per person per year. This is significantly lower than in the US, UK, and other Western European countries, where consumption exceeds 10 kg per person annually. Southeast Asian nations have a higher consumption rate as well, with more than 4.25 kg per person per year. This data highlights the stark differences in biscuit consumption across various regions by Ahmad and Ahmed (3).

*Moringa oleifera*, commonly known as the drumstick tree, horseradish tree, cabbage tree, or miracle tree, is native to India but widely distributed across regions such as Ethiopia, the Pacific Islands, Sudan, the Caribbean, the Philippines, South Africa, Florida, Asia, and Latin America (4). The species is recognized for its nutritional benefits and is promoted by the World Health Organization as a sustainable food source to combat malnutrition. The leaves, which can be consumed fresh or dried using cost-effective solar drying methods, are rich in essential nutrients, including fiber, vitamins A, B, and C, as well as minerals such as magnesium, iron, and calcium. Notably, they exhibit a high protein content, surpassing many legumes, and are abundant in antioxidants, making them a valuable dietary supplement (5).

Jackfruit (*Artocarpus heterophyllus* Lam.) is a tree-borne fruit predominantly cultivated in Thailand, Bangladesh, and India (6). Jackfruit seeds, a significant by-product, constitute over 15% of the fruit’s total weight (7). Fresh seeds are brown, round to oval in shape, measuring 2–4 cm in length and 1.5–2.5 cm in thickness. They are rich in essential nutrients, including starch, protein, and minerals (8). However, limited awareness regarding their utilization leads to substantial wastage annually (9). Processing jackfruit seeds into flour extends their shelf life and enables their incorporation into various food products, either independently or blended with other grain flours, without significantly altering the functional and sensory attributes of the final products, such as cakes, bread, and biscuits. Jackfruit seed consumption offers metabolic benefits, exhibits anti-carcinogenic properties, and protects against skin aging. Additionally, the seeds contain bioactive compounds, including pectin and vitamins A, B, and C, which contribute to pancreatic health and blood purification (10). They are also recognized as a natural source of culinary additives and functional ingredients. Given their high protein content, jackfruit seeds have potential applications as a protein source, particularly in addressing protein-energy malnutrition (PEM) (11) while also aiding digestion (12). The balanced composition of proteins and carbohydrates in jackfruit seed flour has facilitated its use in bakery product fortification.

There is a growing need to partially substitute refined wheat flour-based biscuits with natural, fiber and protein rich ingredients without significantly altering taste. The biscuit industry, a major segment of the processed food market, is rapidly evolving due to advancements in nutraceuticals and new product innovations. Consumer concerns about quality and nutritional content are driving this shift. As the market responds to these demands, incorporating healthier, nutrient-dense ingredients is becoming essential to meet changing consumer preferences and improve the overall nutritional profile of biscuit products. A cost-effective and suitable protein source is needed as a food fortifier to combat malnutrition caused by unbalanced diets in developing countries.

RSM is widely used for modelling and optimising food processing parameters; however, its effectiveness depends on the accuracy of polynomial fitting and local optimisation strategies. In recent years, metaheuristic optimisation algorithms have attracted attention for overcoming these limitations by enabling global search capabilities. Among these, the Grey GWO algorithm, inspired by the social hierarchy and hunting behaviour of grey wolves, has shown strong performance in optimising nonlinear and multi-response systems. Integrating GWO with RSM-derived polynomial models provides a hybrid optimisation framework that enhances solution robustness, avoids local optima, and improves the reliability of optimal process parameter selection (13). Therefore, the present study was undertaken with the primary objective of systematically evaluating the interactive effects of *Moringa oleifera* leaf powder (MOLP) and specific baking conditions temperature and time on the physical and sensory attributes of fortified biscuits. By employing Response Surface Methodology (RSM), this research aimed to determine the optimal substitution levels of MOLP and jackfruit seed powder (JSP) required to maximize nutritional density while preserving the structural integrity and consumer acceptability of the product. Furthermore, a significant objective of this work was to integrate the Grey Wolf Optimization (GWO) algorithm as a robust metaheuristic tool to identify global optima, thereby addressing the limitations of traditional numerical optimization and enhancing the reliability of the process parameters. Ultimately, the study sought to validate these mathematical models through experimental trials to establish a scientifically sound framework for the development of functional, underutilized plant-based bakery products. The novelty of this research lies in its integrated approach to functional food design. By combining the nutritional density of *Moringa oleifera* with the starch-rich properties of jackfruit seed powder, the study addresses the challenge of fortifying bakery products without compromising their structural integrity.

## 2 Materials and methods

### 2.1 Procurement of raw materials

Biscuits were formulated using wheat flour (WF), sugar powder, moringa leaf powder (MOLP), jackfruit seed powder (JSP), ghee, baking powder, and milk. Moringa leaf and jackfruit seed powders were procured from a certified supplier, while other ingredients were sourced from local retailers. Flour sample was stored at −20 °C and used after proper thawing of 2 h at room temperature. All the chemicals used were of analytical grade. The research was conducted at the Department of Processing and Food Engineering, College of Agricultural Engineering & Technology, Dr. Rajendra Prasad Central Agricultural University, Pusa, Samastipur, Bihar.

### 2.2 Physicochemical analysis

Parameters like protein content, crude fat, ash content, moisture content, carbohydrate content and sugar content for both dough and the developed biscuit were analysed as per approved methods of American Association of Cereal Chemists (AACC 2000).

### 2.3 Experimental design for the development of biscuits

The optimization of biscuit formulation was conducted using Response Surface Methodology (RSM) with Design-Expert 10 software. The experimental design involved selecting variable levels, fitting mathematical models, and determining optimal process conditions to maximize desired responses. RSM, a statistical technique, was employed to design experiments that provide relevant information efficiently while minimizing both cost and time. The inclusion levels were selected based on preliminary trials and established literature to ensure a balance between enhanced nutritional density and the preservation of the gluten-starch network (5, 14, 15). A Box–Behnken design (BBD) was used to examine the effects of three independent variables: moringa leaf powder (MOLP) (10%–30%), baking temperature (170 °C–200 °C), and baking time (10–14 min) on six dependent response variables: water activity (aw), spread ratio, colour index, hardness (N), baking weight loss (%), and sensory overall acceptability. These independent variables were designated as A (MOLP %), B (baking temperature, °C), and C (baking time, min). To ensure high experimental reliability and minimize the impact of random error, all 17 treatment combinations generated by the Box–Behnken design were executed in triplicate. The response values used for model fitting and numerical optimization were the calculated means of these three independent trials.

The relationship between independent and response variables was modeled using a second-order polynomial Equation 1:


$$\begin{array}{l} Y=\beta_{o}+\beta ₁A+\beta ₂B+\beta ₃C+\beta ₁₂\mathrm{AB}+\beta ₁₃\mathrm{AC}\\ +\beta ₂₃\mathrm{BC}+\beta ₁₁A^{2}+\beta ₂₂B^{2}+\beta ₃₃C^{2} \end{array}$$ (1)

Where Y represents the predicted response, β_0_ is the intercept, β_1_, β_2_, β_3_ are linear coefficients, β_12_, β_13_, β_23_ are interaction coefficients, and β_11_, β_22_, β_33_ are quadratic coefficients. Regression analysis was performed to optimize the response variables and determine the significance of each factor (16). The experimental design included 17 treatment combinations, generated using BBD, to evaluate the combined effects of the variables. The formulation ratios for MOLP, jackfruit seed powder (JSP), and wheat flour (WF) were 10:30:60, 20:20:60, and 30:10:60. Each experimental run used a 100 g sample, and the process conditions were tested at three levels for each variable: MOLP (10, 20, 30%), baking temperature (170 °C, 185 °C, 200 °C), and baking time (10, 12, 14 min).

### 2.4 Determination of dependent variables

#### 2.4.1 Water activity (aw)

Biscuit were ground and sieved to a uniform particle size of 0.8 mm using a laboratory mill before water activity (aw) analysis. The measurement was conducted at 25 °C using a digital water activity meter (Rotronic HygroPalm), which determines vapour pressure relative to pure water. The sample was enclosed in a sealed chamber until equilibrium was achieved with the headspace, and the water activity was recorded.

#### 2.4.2 Spread ratio

The spread ratio of cookies was determined by measuring their diameter and thickness. The diameter was obtained by placing seven cookies edge to edge in a straight line and recording the total length. The cookies were then rearranged and re-measured to ensure accuracy. Thickness was measured by stacking cookies on top of each other and averaging the total height. The spread ratio was calculated using the Equation 2:


$$\text{Spread Ratio}=\frac{\text{Diameter}(\mathrm{mm})}{\text{Thickness}(\mathrm{mm})}$$ (2)

#### 2.4.3 Colour index

Sample colour characteristics were assessed using the CIELab colorimetric system. Standardized digital imagery was obtained using a calibrated high-resolution camera under controlled illumination. Adobe Photoshop software was employed to analyze the images, with L*, a*, and b* values extracted from three separate areas per image. These parameters quantify lightness (L*), red-green balance (a*), and blue-yellow balance (b*). Results were reported as averages with standard deviations based on the triplicate colour extractions. It was calculated using Equation 3 given by Pathare, Opara and Said, (2013) (17).


$$E=\sqrt{L^{2}+a^{2}+b^{2}}$$ (3)

#### 2.4.4 Hardness (N)

The hardness of the formulated biscuits was determined using a Texture Analyzer (Stable Microsystems, Model TA-HDi, Surrey, UK), equipped with a 50 kg load cell. The test was conducted by recording the maximum force required to fracture the biscuits, providing an objective measure of hardness. The crosshead speed was maintained at 1.66 mm/s during the pre-test phase, 1.66 mm/s during compression, and 2.00 mm/s in the post-test phase.

#### 2.4.5 Baking weight loss (BWL) (%)

BLW is a quantitative measure of the mass reduction in a dough piece during the thermal process, primarily resulting from the evaporation of moisture and the thermal decomposition of leavening agents. It was calculated using Equation 4, based on formula given by Sevimli et al., (2005) (18):


$$\mathrm{BWL}\%=\frac{\left(\text{Initial weight}-\text{Final weight}\right)}{\text{Initial weight}}\times 100$$ (4)

#### 2.4.6 Sensory overall acceptability

Sensory evaluation of the developed biscuits was performed to determine their overall acceptability, appearance, colour, texture, and taste. A trained panel consisting of 25 members (comprising faculty and research scholars from the Department of Processing and Food Engineering, Dr. RPCAU, Pusa) was selected for the study. The panelists were pre-screened for their familiarity with fortified bakery products and underwent orientation sessions to standardize the use of the nine-point hedonic scale, where 1 represented “extreme dislike” and 9 represented “extreme likeness.” Evaluations were conducted in a controlled sensory laboratory environment with individual booths to prevent bias, and samples were presented in a randomized order.”

### 2.5 Biscuit preparation

Biscuit dough was prepared according to AACC method using different combinations of process parameters presented in Table 1. The biscuit preparation process began with weighing the ingredients according to the formulation. The flours, including moringa leaf powder, jackfruit seed powder, and wheat flour, were sieved to ensure uniform particle size. The formulation of the biscuit dough was standardized through a series of preliminary trials to ensure consistent structural and sensory baselines across all experimental treatments. For every 100 g of the composite flour blend consisting of varying ratios of wheat flour, *moringa* leaf powder, and jackfruit seed powder the levels of non-variable ingredients were maintained as follows: 30 g of sugar powder, 30 g of milk, 25 g of ghee, 7 g of milk powder, and 3 g of baking powder. The dry ingredients (milk, sugar, baking powder, and milk powder) were then combined with ghee and milk to form a homogeneous mixture. The dough was prepared by thorough mixing and allowed to rest in a freezer at −18 °C for 30 min to enhance texture and handling properties. The rested dough was then molded and baked at temperatures ranging from 170 °C to 200 °C. To ensure microbiological safety and hygiene, cookies were prepared under strict Good Manufacturing Practices (GMP), and immediately upon cooling, they were sealed in food-grade polyethylene pouches to prevent any post-processing contamination until the time of sensory analysis.

**Table 1:** Independent variables and their respective variation levels.

Treatments	A	B	C	Moringa leaf powder (%)	Baking temperature (°C)	Baking time (Min)
1	1	−1	0	30	170	12
2	−1	0	−1	10	185	10
3	0	1	−1	20	200	10
4	0	0	0	20	185	12
5	0	1	1	20	200	14
6	−1	1	0	10	200	12
7	0	0	0	20	185	12
8	−1	0	−1	10	185	14
9	−1	−1	0	10	170	12
10	0	−1	1	20	170	14
11	0	0	0	20	185	12
12	1	0	1	30	185	14
13	1	0	−1	30	185	10
14	0	0	0	20	185	12
15	1	1	0	30	200	12
16	0	0	0	20	185	12
17	0	−1	−1	20	170	10

### 2.6 Statistical analysis

The experimental data underwent statistical analysis using Design Expert 10 software (State Ease Inc., Minneapolis, USA) to perform analysis of variance (ANOVA) and develop regression models. Second-order polynomial equations were fitted to the data and evaluated for statistical significance. Model validity was assessed through comprehensive model analysis and determination of the coefficient of determination (*R*^2^), which quantifies the ratio of explained variation to total variation, thereby indicating model fitness (19).

### 2.7 Analysis of data

A multiple regression equation was employed to establish model fit, as recommended by (20). Response surface mapping was executed using Design Expert 10 software. Contour plots were generated as functions of two variables while maintaining the third variable at its optimal level to visualize the interaction effects on the response parameters.

### 2.8 Grey wolf optimization (GWO) for model optimization

GWO, a metaheuristic optimisation technique, was proposed by (21), drawing inspiration from the predatory behaviours of grey wolf packs. Due to its simplicity, few control parameters, and strong global search capability, GWO has been increasingly applied in food engineering and process optimisation problem (22).

In the GWO algorithm, the population is divided into four hierarchical groups: alpha (*α*), beta (*β*), delta (*δ*), and omega (*ω*). The α, β, and δ wolves represent the three best candidate solutions, while the remaining wolves update their positions based on these leaders. The hunting behaviour is mathematically modelled by encircling, searching for, and attacking the prey, which corresponds to the optimal solution (23).

The encircling behaviour of grey wolves is defined as shown in Equations (5 and 6). Here, $\vec{X}$ represents the position vector of a grey wolf, while ${\vec{X}}_{p}$ denotes the position of the prey (i.e., the best solution). The coefficient vectors $\vec{A}$ and $\vec{C}$ are defined as given in Equation (7). In Equation (7), $\vec{r_{1}}$ and $\vec{r_{2}}$ [0,1] are random vectors uniformly distributed in the interval [0,1], and the parameter *α* decreases linearly from 2 to 0 throughout the iterations in order to balance exploration and exploitation. Since the exact position of the prey is unknown, the positions of the wolves are updated according to the three best solutions (α, *β*, and *δ*) as expressed in Equation (8).


$$\vec{D}=|\vec{C}\cdot {\vec{X}}_{p}\;-\vec{X}\;|$$ (5)

$$\vec{X}\;(t+1)={\vec{X}}_{p}-\vec{A}\cdot \vec{D}$$ (6)

$$\vec{A}=2a.\vec{r_{1}}-a,\vec{C}=2.\vec{r_{2}}$$ (7)

$$\vec{X}\;(t+1)=\frac{{\vec{X}}_{\alpha }+{\vec{X}}_{\beta }+{\vec{X}}_{\delta }}{3}$$ (8)

In this study, GWO was used to optimise the process variables by maximising the overall desirability function derived from multiple response surface models. The performance of the GWO algorithm depends on the appropriate selection of population size, number of iterations, and control parameters. In this study, the hyperparameters were determined based on ranges recommended in the literature and preliminary trial studies. The population size was set to 25, the number of iterations to 150, and the parameter a was linearly decreased from 2 to 0 throughout the iterations. Random vectors were generated within the interval [0, 1]. Additionally, the Derringer–Suich desirability approach was employed to enable the simultaneous optimisation of multiple responses (SR, E, H, SOA, aw, and BWL). This approach is based on calculating the overall desirability value as the geometric mean of the individual desirability functions defined for each response variable, as shown in Equation (9).


$$D={\left(d_{\mathrm{SR}}d_{E}d_{H}d_{\mathrm{SOA}}d_{\mathrm{aw}}d_{\mathrm{BWL}}\right)}^{1/6}$$ (9)

The response variables were classified according to their optimisation objectives: those to be maximised (e.g., Colour Index and Overall Acceptability), minimised (e.g., water activity), or maintained within a specific range (e.g., Spread Ratio, Hardness, and Baking Weight Loss). For each response variable, linear individual desirability functions were defined, and the overall desirability value was calculated using these functions.

In this context, six second-order polynomial models (SR, E, H, SOA, aw, and BWL), obtained through RSM, were integrated into the GWO algorithm in accordance with the Derringer–Suich approach. Consequently, a multi-objective optimisation process was performed by simultaneously considering all response variables.

## 3 Result and discussion

**Figure 1:**
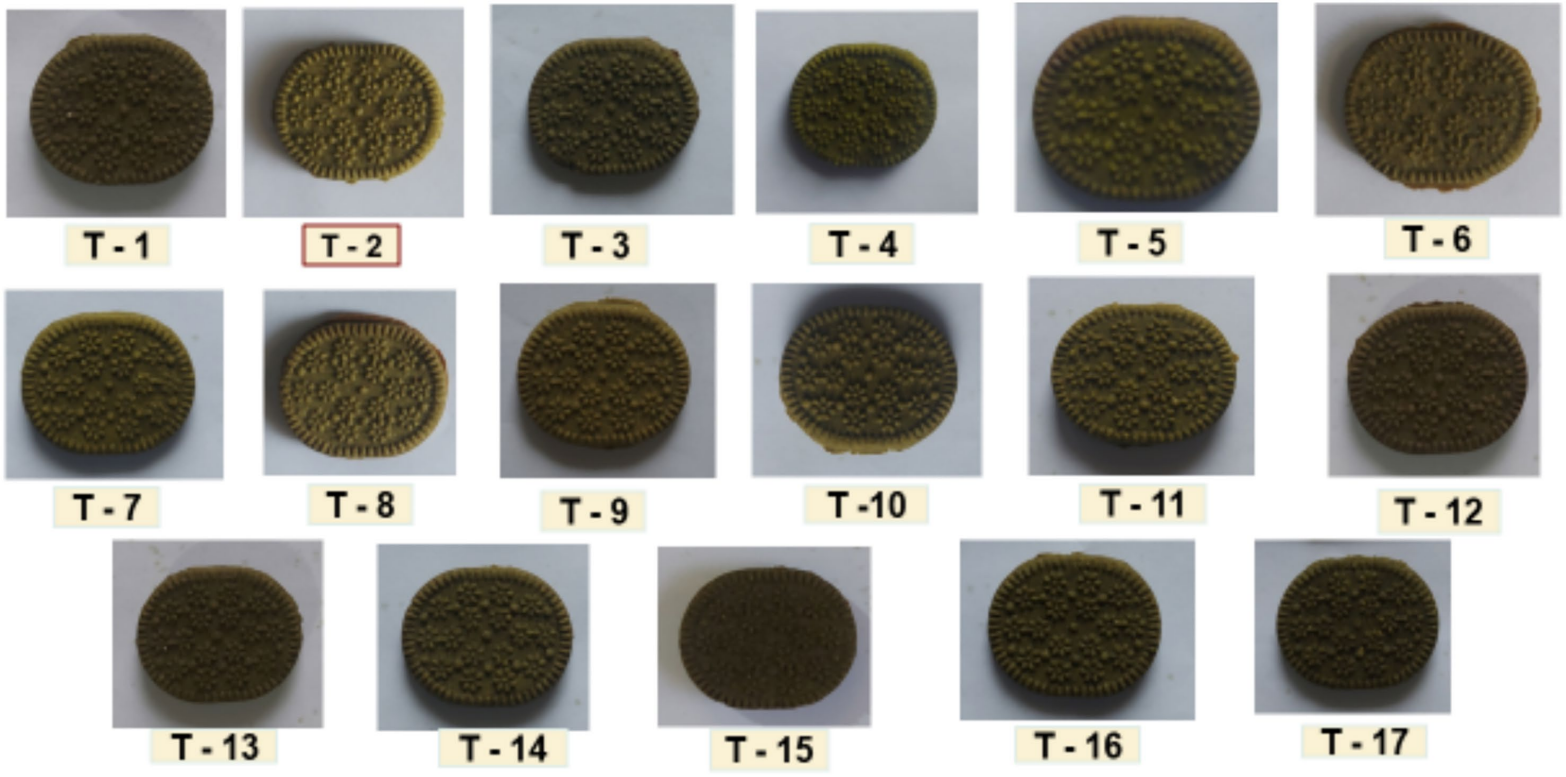
Box–Behnken design matrix showing the effect of independent variables on response variables across 17 experimental trials for biscuit formulation.

This section presents the results of the experimental trials designed using the Box–Behnken Design (BBD) for optimizing the formulation of Moringa and Jackfruit seed-enriched biscuits. The impact of three independent variables Moringa leaf powder percentage, baking temperature, and baking time on key quality parameters was systematically analyzed. The experimental trials yielded 17 treatment combinations, each reflecting a unique set of processing conditions and response outcomes presented in Figure 1.

### 3.1 Effects independent variables on spread ratio

The influence of moringa leaf powder (MLP) percentage, baking temperature, and baking time on the spread ratio of biscuits was evaluated, with results summarized in Table 2. The spread ratio varied from 6.22 to 7.96 under different processing conditions. A second-order polynomial model (Equation 10) was developed to describe the empirical relationship:


$$\begin{array}{l} \text{Spread Ratio}=6.68+0.31A+0.26B–0.48C\\ +0.35\mathrm{AB}+0.16\mathrm{AC}–0.072\mathrm{BC}+0.26A^{2}–0.20B^{2}+0.40C^{2} \end{array}$$ (10)

**Table 2:** Variation in predicted and calculated values of dependent variables as affected by independent variables (Box–Behnken Design Matrix) for different treatment.

Run	Moringa Leaf Powder	Temperature	Time	Spread Ratio (pred.)	Spread Ratio (Exp.)	Colour Index (pred.)	Colour Index (Exp.)	Hardness (pred.)	Hardness (Exp.)	Overall Acceptability(pred.)	Overall Acceptability (Exp)	Water Activity (pred.)	Water Activity (exp.)	Baking Weight Loss (pred.)	Baking Weight Loss (Exp.)
	%	°C	Minutes	SR	SR		E	gm	gm		OA		a_w_		%
1	30	170	12	6.99	6.55	154.33	148.86	2257.51	2235.75	7.8	8.36	0.712	0.71	9.15	9.01
2	10	185	10	7.25	7.91	152.5	155.09	2555.77	2595.98	7.8	7.66	0.682	0.66	7.7	7.01
3	20	200	10	7.56	7.55	161.79	161.01	2501.41	2477.46	7.24	6.80	0.677	0.66	8.28	8.66
4	20	185	12	6.68	6.47	159.02	158.98	2481.49	2498.05	7.24	7.34	0.71	0.70	8.57	8.33
5	20	200	14	6.6	6.71	160.71	161.03	2461.57	2456.96	7.24	7.43	0.743	0.72	8.28	8.19
6	10	200	12	6.37	6.22	158.79	158.94	2555.77	2566.87	7.8	8.06	0.656	0.65	7.41	7.03
7	20	185	12	6.68	7.02	159.02	158.02	2481.49	2487.02	7.24	7.08	0.71	0.67	8.57	8.38
8	10	185	14	6.29	6.37	160.62	162.13	2555.77	2504.87	7.8	7.70	0.682	0.64	7.7	7.54
9	10	170	12	6.37	6.39	154.33	156.87	2555.77	2516.96	7.8	7.70	0.708	0.73	7.41	7.23
10	20	170	14	6.6	6.34	160.81	163.22	2501.41	2486.96	7.24	7.63	0.677	0.67	8.28	8.37
11	20	185	12	6.68	7.09	159.02	156.78	2481.49	2481.57	7.24	7.76	0.71	0.73	8.57	8.94
12	30	185	14	6.91	7.08	155.98	155.86	2257.51	2276.98	7.8	7.76	0.738	0.73	9.44	9.11
13	30	185	10	7.87	7.96	157.14	158.09	2257.51	2201.93	7.8	7.80	0.738	0.73	9.44	9.34
14	20	185	12	6.68	6.23	159.02	161.36	2481.49	2476.82	7.24	7.00	0.71	0.72	8.57	8.47
15	30	200	12	6.99	7.77	158.79	159.90	2257.51	2276.98	7.8	7.80	0.764	0.73	9.15	9.20
16	20	185	12	6.68	6.58	159.02	159.95	2481.49	2463.98	7.24	7.12	0.71	0.70	8.57	8.75
17	20	170	10	7.56	6.89	152.77	154.09	2461.57	2427.77	7.24	7.56	0.743	0.74	8.28	8.22

The model demonstrated a good fit (*R*^2^ = 0.85, *p* < 0.05) and high experimental reliability (CV = 4.89%). Regression analysis (Table 3) indicated that the linear terms of MLP percentage and baking time significantly influenced spread ratio (*p* < 0.05), with baking time exerting a highly significant positive effect (*p* < 0.01). Among the quadratic terms, only baking time was significant (*p* < 0.05), while all interaction effects were non-significant (*p* > 0.05).

**Table 3:** Regression coefficients of fitted second order model of the responses for optimization of developed biscuits and ANOVA of the determined values.

Terms	Regression coefficients					
	Spread ratio (SR)	Colour index (E)	Hardness (N)	Overall acceptability (OA)	Water activity (a_w_)	Baking weight loss (%)
Intercept						
β_0_	6.68	159.02	2481.49	7.24	+0.71	+8.57
Linear						
β_1_	0.31*	−1.29	−149.13**	0.075	0.028**	+0.87**
β_2_	0.26	2.23*	13.85	−0.14	−7.250E-003	+0.031
β_3_	−0.48**	1.74*	2.83	0.086	−1.875E-003	−0.12
Quadratic						
β_11_	+0.26	−2.46*	−74.85**	0.56*	+8.500E-004	−0.17
β_22_	−0.20	−0.42	−7.50	0.18	+1.600E-003	−0.29*
β_33_	+0.40*	1.24	−11.70	−0.067	−9.150E-003	+0.076
Interaction						
β_12_	+0.35	2.24	−2.17	−0.23	+0.026*	+0.097
β_13_	−0.16	−2.32*	41.54*	−0.017	+3.500E-003	+0.042
β_23_	−0.072	−2.28*	19.92*	+0.14	+0.033*	−0.15
R^2^	0.85	0.87	0.99	0.83	0.83	0.94
Mean	6.89	158.25	2437.23	7.56	0.71	8.39
Standard deviation	0.34	0.91	16.77	0.25	0.020	0.24
CV (%)	4.89	1.20	0.69	3.25	2.85	2.90
*p* value (Regression)	0.0263*	0.0196*	0.0001**	0.0461*	0.0437*	0.0015**
*p* value (lack of fit)	0.6504	0.3685	0.1717	0.6308	0.6267	0.6041

CV, coefficient of variation. *Significant at *p* ≤ 0.05; **Significant at *p* ≤ 0.01.

An increase in MLP percentage from 10% to 30% resulted in higher spread ratios, likely due to the dilution of gluten-forming proteins in wheat flour by the gluten-free, fiber-rich MLP, which disrupts gluten network development (24, 25). Additionally, the presence of phytochemicals and natural oils in MLP may reduce dough viscosity, further enhancing spread (26). Baking at 185 °C yielded the highest spread ratios. Lower temperatures (170 °C) slow protein coagulation and starch gelatinization, limiting spread, whereas higher temperatures (200 °C) promote rapid crust formation, restricting dough expansion (27, 28). Baking times of 10–12 min allowed optimal dough flow before setting, while extended times (14 min) reduced spread due to moisture loss and structural solidification. These findings underscore the critical role of ingredient proportions and baking conditions in shaping biscuit geometry and quality, consistent with prior studies (29–31).

### 3.2 Effect of independent variables on colour index

The colour index of biscuits, which reflects surface browning and visual quality, was significantly influenced by variations in moringa leaf powder (MLP) percentage, baking temperature, and baking time. The observed colour index ranged from 148.86 to 163.22 across the design space (Table 2), indicating substantial variation under different combinations of processing parameters.

The second-order polynomial model (Equation 11) demonstrated a strong predictive capability with a coefficient of determination (*R*^2^) of 0.87 and a low coefficient of variation (CV = 1.20%), confirming model adequacy and experimental reliability. Regression analysis (Table 3) revealed that the linear terms of baking temperature (B) and baking time (C) positively influenced the colour index (*p* < 0.05), suggesting that increasing either parameter enhances browning. This effect can be attributed to intensified Maillard and caramelization reactions, which are thermally activated and contribute to colour development during baking (32, 33).


$$\begin{array}{l} \text{Color Index}=159.02–1.29A+2.23B+1.74C+\\ 2.24\mathrm{AB}–2.32\mathrm{AC}–2.28\mathrm{BC}–2.46A^{2}–0.42B^{2}+1.24C^{2} \end{array}$$ (11)

Among interaction terms, both MLP percentage × time (AC) and temperature × time (BC) exhibited significant negative coefficients (*p* < 0.05), indicating that excessive combination of high baking time and temperature or higher MLP content with longer baking may lead to diminishing or even adverse effects on browning (34). This could be due to the antioxidant properties and green pigments of moringa, such as chlorophyll and flavonoids (35), which might interfere with browning reactions or mask Maillard pigments.

**Figure 2:**
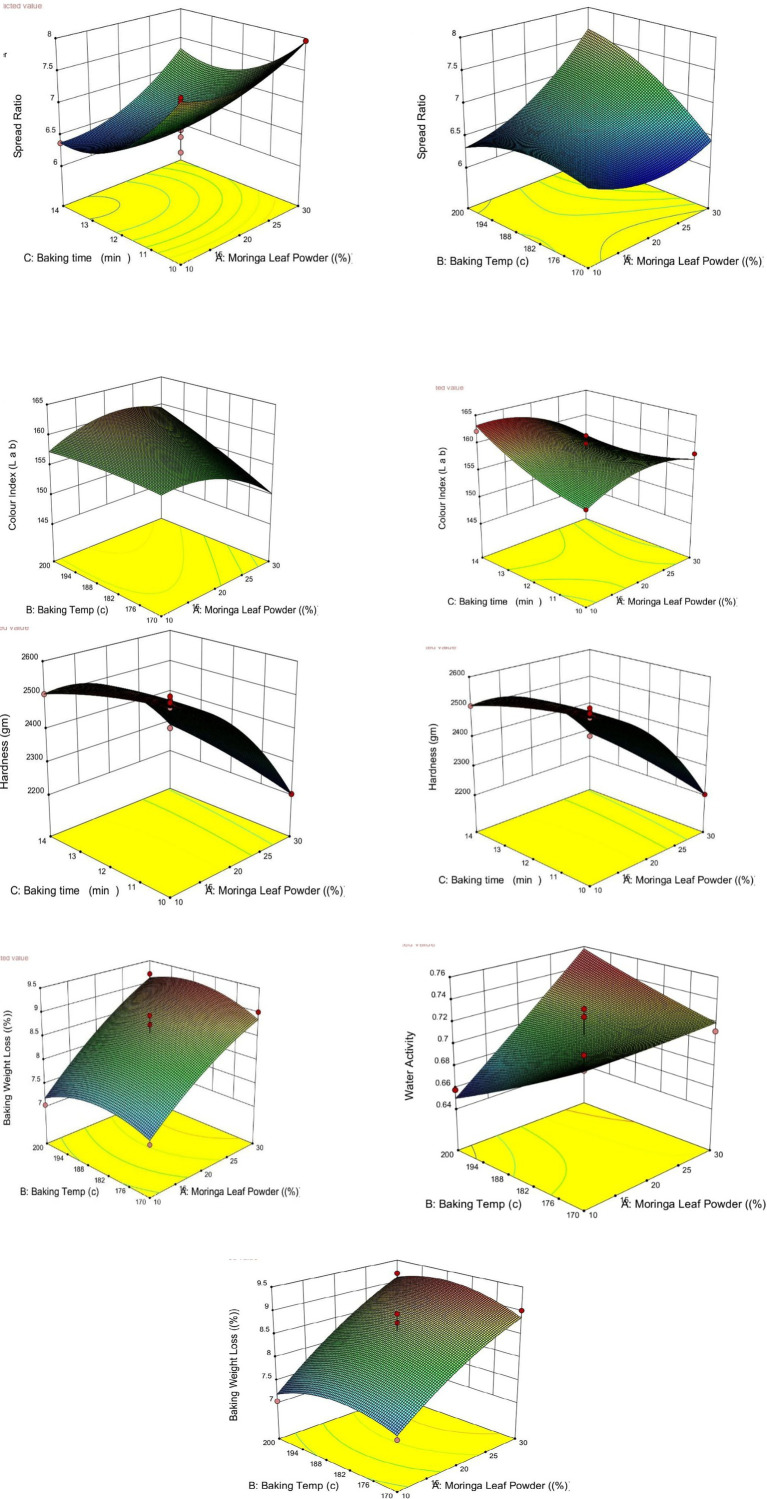
Three-dimensional (3D) response surface plots illustrating the interactive effects of independent variables (MOLP %, baking temperature, and baking time) on critical quality responses including spread ratio, color index, and biscuit hardness.

Interestingly, the quadratic effect of MLP percentage (A^2^) was significant, with a negative coefficient, highlighting a nonlinear relationship wherein colour index initially increased with MLP addition but declined beyond a certain concentration. This aligns with visual observations where higher MLP concentrations can impart a greenish hue, potentially overriding the brown tones from Maillard products and reducing overall colour intensity (15). Response surface plots (Figure 2) further supported these interactions, particularly emphasizing that higher colour index values were achieved at moderate MLP levels and optimal combinations of temperature and time. Excessive values of any one factor led to suboptimal colour development (36). Similar findings were reported by (37–39) for beetroot, Cookies Prepared from Pearl Millet and apple puree, respectively.

### 3.3 Effect of independent variables on hardness

The effect of moringa leaf powder concentration (A), baking temperature (B), and baking time (C) on the hardness of the developed biscuits was studied using response surface methodology. The experimental hardness values ranged from 2201.93 g to 2595.98 g (Table 2). A second-order polynomial model was fitted to the data and was found to be highly adequate with a coefficient of determination (*R*^2^ = 0.99), indicating that 99% of the variation in biscuit hardness was explained by the model a low coefficient of variation (CV = 0.69%), confirming model adequacy and experimental reliability (Table 3). The final regression (Equation 12) in terms of coded variables for significant terms was:


$$\begin{array}{l} \text{Hardness}\;(g)=2481.49–149.13A+13.85B+2.83C–\\ 2.17\mathrm{AB}+41.54\mathrm{AC}–19.92\mathrm{BC}–74.85A^{2}–7.50B^{2}–11.70C^{2} \end{array}$$ (12)

Among the independent variables, the linear effect of moringa leaf powder percentage (A) was found to be highly significant (*p* < 0.01), indicating its strong negative influence on biscuit hardness. This may be attributed to the fibrous and coarse nature of moringa leaf powder, which likely disrupted gluten formation and reduced dough cohesiveness, thereby resulting in softer biscuits (40).

While the linear terms for baking temperature and time (B and C) and all interaction terms (AB, AC, BC) were statistically insignificant at *p* < 0.05, the interaction between baking temperature and baking time (BC) was significant (*p* < 0.05). This indicates that the combined effect of temperature and time plays a notable role in determining the final texture of the biscuit (41), possibly due to its influence on Maillard reactions and internal moisture redistribution (31).

In the quadratic terms, A^2^ was highly significant (*p* < 0.01), suggesting a strong curvilinear relationship between moringa powder concentration and hardness. This implies that beyond a certain level, further increase in moringa content may have a diminishing or opposing effect on hardness (42). The quadratic terms for B^2^ and C^2^ were not significant, indicating relatively linear effects over the studied range.

### 3.4 Effect of independent variables on sensory overall acceptability

The influence of moringa leaf powder concentration (A), baking temperature (B), and baking time (C) on the sensory overall acceptability (SOA) of the developed biscuits was assessed using a second-order polynomial regression model. The observed SOA scores ranged from 6.80 to 8.36 (Table 2) on the 9-point hedonic scale, indicating variations in sensory perception due to formulation and processing conditions. The generated polynomial (Equation 13) for SOA for was


$$\begin{array}{l} \mathrm{SOA}=7.24+0.075A–0.14B+0.086C–0.23\mathrm{AB}\\ –0.017\mathrm{AC}+0.14\mathrm{BC}+0.56A^{2}+0.18B^{2}–0.067C^{2} \end{array}$$ (13)

The model showed acceptable fitness with a coefficient of determination (R^2^ = 0.83) and coefficient of variation (CV) of 3.25% (Table 3), indicating reliable predictive ability with low dispersion of data. The regression analysis revealed that the model as a whole was significant (*p* < 0.05). Among all the terms, only the quadratic term of moringa leaf powder concentration (A^2^) was found to be highly significant (*p* < 0.01). This indicates a curvilinear relationship, suggesting that both very low and very high levels of moringa leaf powder may reduce overall acceptability, while a moderate level enhances it (43). The improvement at moderate levels may be attributed to the unique flavour and colour imparted by moringa (44) and jackfruit seed power, which might become overpowering at higher concentrations.

All other linear, interaction, and quadratic terms were found to be statistically non-significant, indicating a minimal independent or combined influence of baking temperature and time on sensory acceptability within the studied range. The relatively lower significance of these processing factors suggests that consumer perception was predominantly influenced by the formulation component, i.e., moringa content and jackfruit seed power.

### 3.5 Effect of independent variables on water activity (aw)

The effect of moringa leaf powder concentration (A), baking temperature (B), and baking time (C) on the water activity of the developed biscuits was studied using a second-order polynomial regression model. The experimental values of water activity ranged between 0.64 and 0.74 (Table 2), indicating a safe range for microbiological stability in low moisture baked products. The quadratic regression (Equation 14) including significant terms was:


$$\begin{array}{l} \text{Water Activity}=0.71+0.028A–0.00725B\\ –0.001875C+0.026\mathrm{AB}+0.0035\mathrm{AC}+0.033\mathrm{BC}+0.00085A^{2}\\ +0.0016B^{2}–0.00915C^{2} \end{array}$$ (14)

The model was found to be significant (*p* < 0.05), with a coefficient of determination (R^2^ = 0.8331) and a low coefficient of variation (CV = 2.85%) (Table 3), confirming the model’s suitability and precision. Among the variables, the linear term of moringa leaf powder concentration (A) was highly significant (*p* < 0.01), showing a direct influence on increasing water activity. This can be attributed to moringa’s natural hygroscopic properties (45), which can retain moisture and influence the amount of free water in the biscuit matrix.

In addition, the interaction terms AB (moringa × temperature) and BC (temperature × time) were also significant (*p* < 0.05). These interactions suggest that the drying efficiency and moisture migration are significantly influenced by combined effects of formulation and baking conditions (46). At higher moringa leaf power percentage, an increase in temperature may be required to achieve desired moisture reduction, and similarly, the combined effects of temperature and time govern final product dryness (47). All other linear, interaction, and quadratic terms were found to be statistically non-significant and were excluded from the final model equation.

### 3.6 Effect of independent variables on baking weight loss (%)

The influence of moringa leaf powder concentration (A), baking temperature (B), and baking time (C) on the baking weight loss (%) of the developed biscuits was analyzed using a second-order polynomial model. The observed baking weight loss ranged from 7.01% to 9.34% (Table 2), indicating variability depending on formulation and baking conditions. The reduced regression Equation 15, including statistically significant terms, was:


$$\begin{array}{l} \text{Baking Weight Loss}\;(\%)=8.57+0.87A+0.031B–0.12C\\ +0.097\mathrm{AB}+0.042\mathrm{AC}–0.15\mathrm{BC}–0.17A^{2}–0.29B^{2}+0.076C^{2} \end{array}$$ (15)

The model was found to be statistically significant (*p* < 0.05) with a high coefficient of determination (*R*^2^ = 0.9422) and a low coefficient of variation (CV = 2.90%) (Table 3), demonstrating the model’s reliability and precision in explaining the variation in baking weight loss. Among the predictors, the linear term of moringa leaf powder concentration (A) was highly significant (*p* < 0.01), indicating a strong positive influence on baking weight loss. This suggests that increasing moringa leaf powder levels leads to higher moisture evaporation during baking, possibly due to the porous structure and hygroscopic nature of moringa that promotes moisture loss (48).

In the quadratic terms, baking temperature squared (B^2^) was significant (*p* < 0.05). This highlights the non-linear relationship between temperature and moisture loss (49), where excessive temperature may eventually reduce further evaporation due to crust formation or drying saturation (49). All other linear, interaction, and quadratic terms were statistically non-significant.

### 3.7 Numerical optimization and model verification

Table 4 presents the constraints applied to the independent variables (blend ratio, baking temperature, and baking time) and the desired goals for each response during numerical optimization. All parameters were set within range, except for colour index and overall acceptability, which were maximized, and water activity, which was minimized. The optimum conditions obtained were 10% Moringa leaf powder, 193.68 °C baking temperature, and 13.97 min baking time. Under these settings, the model predicted desirable response values, achieving an overall desirability of 0.828.

**Table 4:** Constraints applied to the processing parameters and responses for numerical optimization parameters.

Parameters	Conditions	Lower limit	Upper limit	Importance^a^	Optimum value^b^
Blend ratio (%MOLP)	In range	10	30	3	10
Baking temperature (o C)	In range	170	200	3	193.68
Baking time (min)	In range	10	14	3	13.97
Responses					
Spread ratio (SR)	In range	6.22	7.96	3	6.220
Colour index (E)	Maximum	148.86	163.22	3	161.68
Hardness (g)	In range	2201.93	2595.98	3	2500.58
Sensory overall acceptability (OA)	Maximum	6.8	8.36	3	7.95
Water activity (aw)	Minimum	0.64	0.74	3	0.66
Baking weight loss (%)	In range	7.01	9.34	3	7.23

a The value of importance is as per the default setting of the software, b.

b The desirability for this result was 0.828.

Table 5 presents the model validation results comparing RSM-predicted values with experimental means. Most responses, including spread ratio, sensory overall acceptability, and water activity, showed no significant difference (*p* > 0.05), indicating strong model reliability. However, significant deviations were observed in colour index (*p* = 0.02), hardness (*p* = 0.04), and baking weight loss (*p* = 0.003), suggesting that while the model generally performed well, certain responses may be influenced by factors not fully captured in the model or inherent variability in physical properties during baking.

**Table 5:** Verification of the models by comparing the experimental values with the predicted values Response.

Responses	Predicted value	Actual value^a^ ±SD	Standard error	Mean difference	T statistic	*p*-value	95% CI for difference	
							Upper limit	Lower limit
Spread Ratio (SR)	6.22	6.40 ± 0.30	0.17	0.18	1.03	0.41	7.16	5.63
Colour Index (E)	161.68	150.91 ± 2.69	1.55	−10.77	−6.92	0.02	157.60	144.22*
Hardness (g)	2500.58	2294.79 ± 81.56	47.09	−205.71	−4.36	0.04	2497.41	2092.17*
Sensory Overall Acceptability (OA)	7.95	8.28 ± 0.18	0.10	0.33	3.14	0.08	8.73	7.83
Water Activity (aw)	0.66	0.67 ± 0.04	0.02	0.01	0.22	0.84	0.77	0.57
Baking weight loss (%)	7.23	8.51 ± 0.12	0.07	1.28	17.58	0.003	8.83	8.20**

a Mean of three replication.

*Significant at *p* ≤ 0.05; **Significant at *p* ≤ 0.01.

### 3.8 GWO-based optimization of RSM models

While conventional RSM-based numerical optimisation provides efficient local solutions, its performance may be limited by the quadratic nature of the fitted models and the presence of multiple conflicting responses. Therefore, integrating GWO with RSM enables a robust global search across the design space, allowing simultaneous optimisation of multiple quality attributes through a composite desirability function.

The GWO algorithm was applied to maximise the overall desirability (D), which integrates spread ratio, colour index, hardness, sensory overall acceptability, water activity, and baking weight loss into a single objective. The optimisation process not only identifies the optimal processing conditions but also provides insight into convergence behaviour, trade-offs among responses, and boundary-driven solution characteristics.

The final desirability value reaches approximately D = 0.882. The optimal processing conditions were A = 10.00% MOLP, B = 193.96 °C, and C = 14.00 min.

Under these conditions, the predicted responses were spread ratio = 6.22, colour index = 161.63, hardness = 2524.15 g, overall acceptability = 7.96, water activity = 0.67, and baking weight loss = 7.21%. All response values fall within their respective acceptable or target ranges, confirming the feasibility and robustness of the obtained solution.

**Figure 3:**
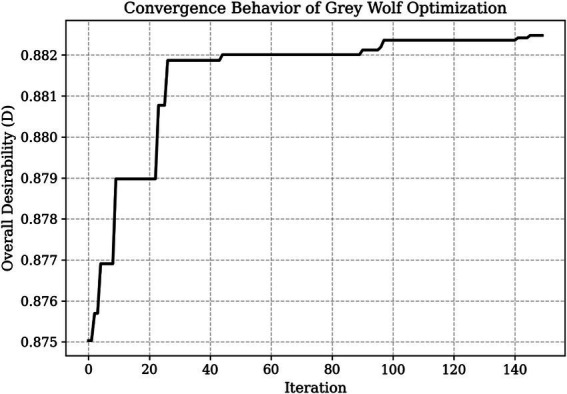
This figure illustrates the convergence behaviour of the GWO algorithm in terms of the overall desirability function (D).

The convergence behaviour of the GWO algorithm with respect to overall desirability is shown in Figure 3. The optimisation process shows a rapid increase in desirability during the initial iterations, indicating effective global exploration of the search space. Within approximately the first 20–30 iterations, the algorithm identifies a promising region, followed by a gradual improvement phase with smaller increments. After about 90–100 iterations, the desirability value stabilises, indicating convergence to a near-global optimum.

The smooth convergence model, which does not exhibit oscillatory behaviour, demonstrates the numerical stability and robustness of the GWO algorithm when applied to response surface-based optimisation problems. The final desirability value reaches approximately D = 0.882, confirming that multiple quality objectives can be successfully met simultaneously.

Figure 3 presents the combined optimisation, showing both the overall desirability (D) and the individual desirability components for each response. Unlike single-objective optimisation, this study uses a composite desirability function integrating six quality responses: spread rate, colour index, hardness, overall acceptability, water activity, and baking weight loss. Thus, the optimisation process aims to improve aggregate process performance rather than independently maximising individual responses.

**Figure 4:**
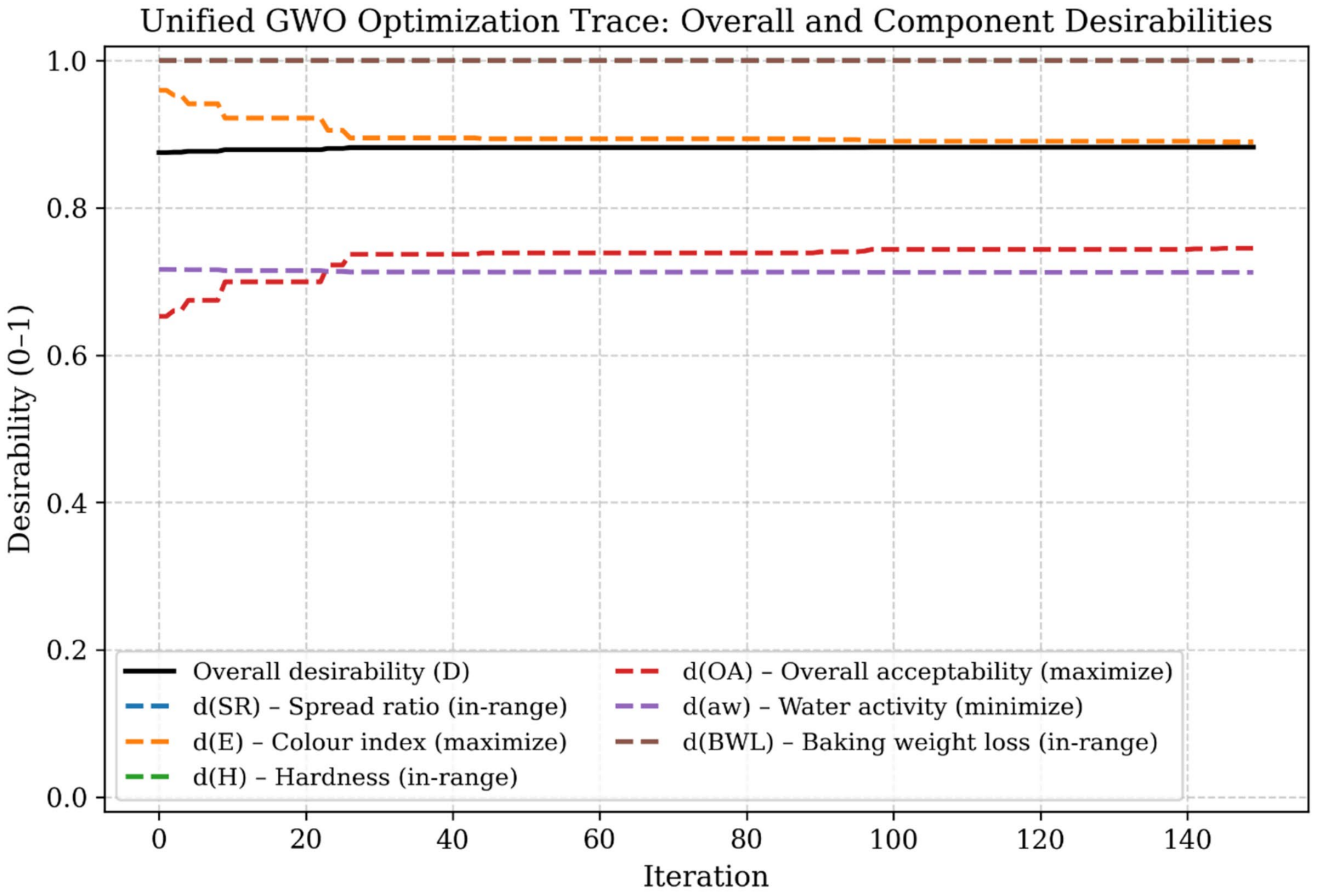
This figure presents the unified optimisation trace, showing both the overall desirability (D) and the individual desirability components for each response.

As shown in Figure 4, the desirability components for baking weight loss and hardness rapidly reach values close to unity, indicating that these constraints are readily satisfied within the feasible design space. In contrast, the desirability functions for colour index, overall acceptability, and water activity play a more critical role in guiding the optimisation trajectory. Incremental increases in these components directly drive the improvement of overall desirability.

This behaviour highlights the trade-off inherent in the optimisation problem and confirms that the final solution represents a balanced compromise between competing quality objectives, rather than maximising a single response.

**Figure 5:**
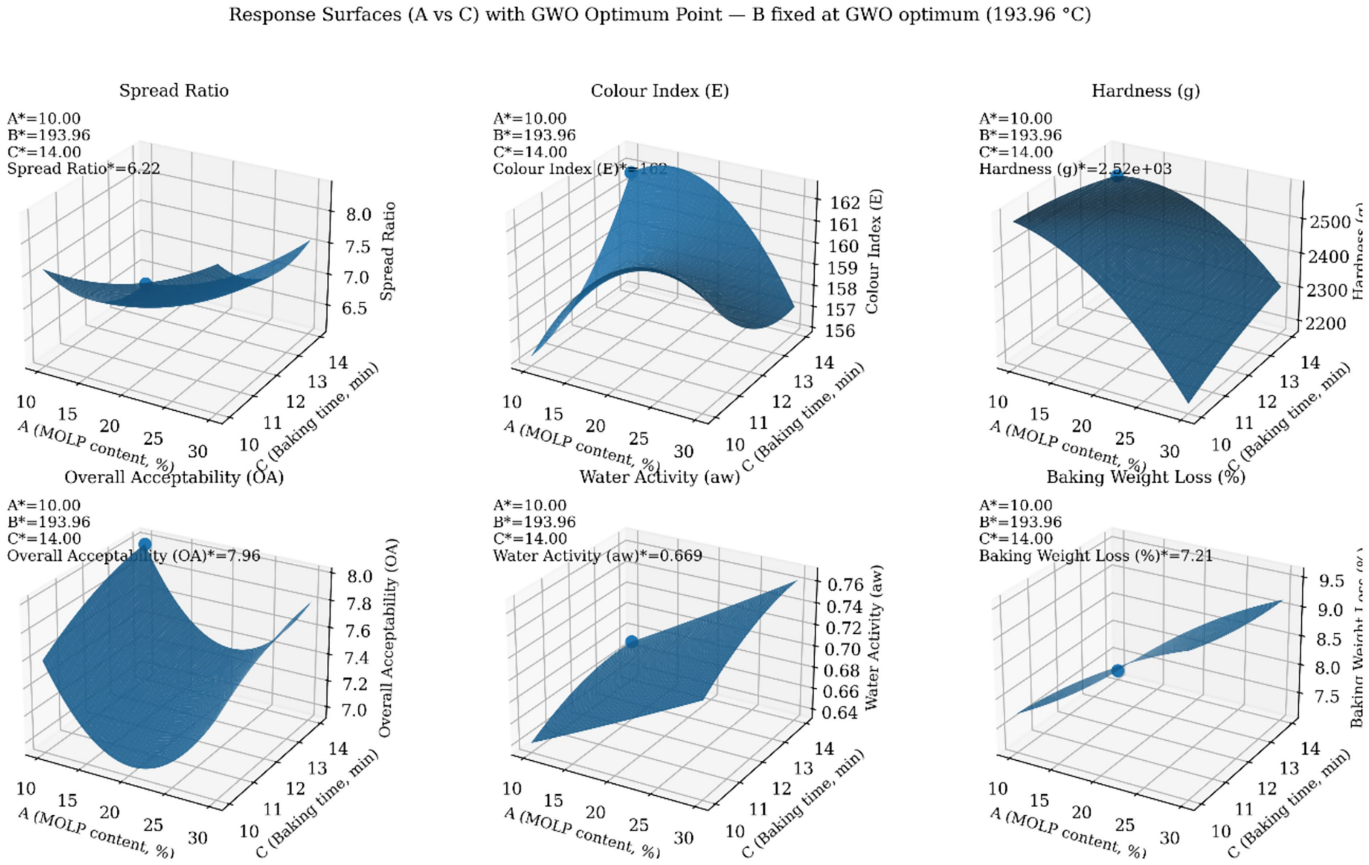
Response surface analysis of quality attributes with the GWO optimum highlighted.

Figure 5 shows the response surface plots for spread ratio, colour index, hardness, overall acceptability, water activity, and baking weight loss as functions of MOLP content (A) and baking time (C), with baking temperature (B) fixed at the value corresponding to the GWO optimum (193.96 °C). The GWO-derived optimal point is superimposed on each surface to illustrate its position relative to the response landscapes.

The response surfaces indicate that MOLP content and baking time have pronounced and often monotonic effects on several quality attributes. Notably, the optimal solution is located near the minimum MOLP content and maximum baking time boundaries, consistent with the desirability-based optimisation results. For desirable responses such as colour index and overall acceptability, the optimal point lies within elevated regions of the surfaces, while for constrained responses such as water activity and baking weight loss, the optimal solution remains within acceptable ranges rather than at extreme values.

The consistent placement of the GWO optimum across all response surfaces confirms the compatibility between the quadratic RSM models and the metaheuristic optimisation framework. Furthermore, the results show that the identified optimum represents a balanced compromise among competing quality attributes rather than an extreme optimisation of a single response.

Overall, the GWO-based optimisation successfully improved the predictive RSM models by enabling a global search of the design space and resolving trade-offs among multiple quality responses. The convergence behaviour, desirability evolution, and response surface analyses consistently confirm that the identified optimum is a robust and physically meaningful solution. These results demonstrate that integrating GWO with RSM is an effective strategy for multi-response optimisation of baking process parameters, providing both numerical efficiency and practical interpretability.

## 4 Conclusion

This study successfully optimized the process parameters for the development of functional biscuits enriched with the addition of *Moringa oleifera* leaf powder and jack fruit seed powder using Response Surface Methodology (RSM). Within the scope of the research, a new product formulation with high nutritional value, sensory acceptance and shelf-life advantage was obtained by integrating plant-derived functional ingredients into conventional bakery products.

The optimum product formulation was determined as 10% Moringa oleifera leaf powder, 30% jack fruit seed powder, 193.68 °C baking temperature and 13.97 min baking time. The biscuits obtained under these conditions exhibited optimal properties in terms of quality criteria such as desired spreading rate, increased color index, sensory acceptance hardness, low water activity and weight loss due to controlled baking. In addition, the sensory analysis results of the products were evaluated positively in terms of consumer appreciation; high scores were obtained especially in taste, texture and general acceptability parameters.

Model validation analyses confirmed the reliability and predictive accuracy of the optimized formulation; the experimentally obtained responses were found to be close to the values predicted by the model without showing any statistically significant difference. This increases the usability of the developed model in industrial applications.

In addition to conventional RSM optimisation, integrating the Grey Wolf Optimisation (GWO) algorithm enabled an efficient global search of the design space using a composite desirability function. The GWO-based optimisation showed that *Moringa oleifera* leaf powder content and baking time converged at their experimental boundaries, reflecting their monotonic influence on multiple quality responses rather than a limitation of the optimisation method. This boundary-dominated solution highlights the importance of metaheuristic optimisation in resolving multi-response trade-offs and identifying robust operating conditions for functional bakery products.

As a result, the optimized biscuit formulation provides both economic and health advantages compared to traditional products, thus creating an important alternative in terms of functional food design. Evaluation of plant sources such as *Moringa oleifera* and jack fruit seeds as sustainable and nutritional additives offers significant potential in future studies on the development of functional bakery products.

## Data Availability

The raw data supporting the conclusions of this article will be made available by the authors, without undue reservation.
